# Comparison of the Kynurenine/Tryptophan Ratio with the Beck Suicide Intent Scale in Patients Admitted to the Emergency Department Due to Suicide Attempt

**DOI:** 10.3390/jcm14196859

**Published:** 2025-09-28

**Authors:** Osman Lütfi Demirci, Emin Fatih Vişneci, Demet Acar, Ümmügülsüm Can, Fatih Cemal Tekin, Mehmet Gül, Berke Yıldırım

**Affiliations:** Emergency Department, Konya City Hospital, Konya 42020, Turkey; drfatihvisneci@hotmail.com (E.F.V.); dr_demetacar@hotmail.com (D.A.); cangulsum@yahoo.com (Ü.C.); fatihcemaltekin@gmail.com (F.C.T.); mehmetgul156@yahoo.com (M.G.); brkeyildirim@gmail.com (B.Y.)

**Keywords:** emergency department, tryptophan, kynurenine, KYN/TRP ratio, suicide, Beck Suicide Intent Scale, biomarker

## Abstract

**Objective:** Suicide is a major public health problem with multiple biological and psychosocial determinants. Although the kynurenine/tryptophan (KYN/TRP) pathway has been implicated in the neurobiology of suicidal behavior, clinical findings remain inconsistent. This study aimed to evaluate serum tryptophan, kynurenine, and the KYN/TRP ratio in patients presenting to the emergency department after a suicide attempt and to examine their association with suicide risk. **Methods:** This prospective, cross-sectional, and comparative study was conducted between November 2024 and June 2025 in the Emergency Department of Konya City Hospital. A total of 120 participants were enrolled, including 60 suicide attempt cases and 60 healthy controls. Serum tryptophan and kynurenine levels were measured using the ELISA method, and the KYN/TRP ratio was calculated in molar units. The Beck Suicide Intent Scale (SIS) was administered to the case group. Group comparisons and correlation analyses were performed using appropriate statistical tests, and effect sizes with 95% confidence intervals were reported. **Results:** Compared with controls, patients showed significantly lower levels of tryptophan (median 35.4 vs. 54.4; *p* = 0.002), kynurenine (median 1534.5 vs. 2384.0; *p* < 0.001), and the KYN/TRP ratio (40.9 ± 16.2 vs. 48.8 ± 20.8; *p* = 0.02). No significant correlations were found between SIS scores and tryptophan (*p* = 0.180), kynurenine (*p* = 0.668), or the KYN/TRP ratio (*p* = 0.246). Subgroup analyses based on psychiatric history or psychiatric consultation recommendations also revealed no significant differences. **Conclusions:** Serum tryptophan, kynurenine, and the KYN/TRP ratio were significantly reduced in patients with suicide attempts compared to healthy controls. However, these biochemical parameters were not associated with SIS scores. Our findings suggest that tryptophan, kynurenine, and the KYN/TRP ratio may serve as complementary biomarkers but cannot replace clinical and psychometric assessments. Larger, multicenter, and longitudinal studies are needed to clarify their potential clinical value.

## 1. Introduction

Suicide is defined as the deliberate and intentional act of ending one’s own life, representing a fatal behavior that results from the complex interplay of numerous biological and psychosocial factors [1]. It often arises from the cumulative effects of psychosocial stressors such as helplessness, hopelessness, loneliness, and inadequate coping mechanisms. Psychiatric disorders—particularly major depressive disorder, bipolar disorder, schizophrenia, and substance use disorders—are among the most prominent determinants of suicidal behavior [1,2]. Furthermore, adverse experiences such as childhood trauma, sexual or physical abuse, family conflict, economic hardship, social isolation, and societal pressures significantly increase the risk of suicide [3,4].

According to the most recent data from the World Health Organization (WHO), approximately 703,000 individuals die by suicide each year, equating to one death every 40 s [5]. The incidence of non-fatal suicide attempts is estimated to be 10–20 times higher, representing a growing public health concern, particularly among adolescents and young adults [4,6]. Globally, suicide is the second leading cause of death among individuals aged 15–29 years, surpassed only by unintentional injuries [5]. This highlights the urgent need for early detection and effective intervention strategies.

At present, suicide risk assessment in healthcare systems relies primarily on psychometric scales and clinical evaluations. However, these subjective approaches carry the risk of misclassification and may delay timely preventive interventions [7]. Incorporating biological risk markers alongside psychological assessments could contribute to more objective risk stratification and allow for more targeted pharmacological and psychosocial interventions [8].

Neurobiological research has implicated several mechanisms in suicidal behavior, including dysregulation of the hypothalamic–pituitary–adrenal (HPA) axis, disturbances in the serotonergic system, and heightened inflammatory responses [6,9]. Tryptophan (Trp), the essential precursor of serotonin synthesis, plays a critical role in mood regulation, impulse control, and decision making; reduced plasma tryptophan levels may adversely affect these processes [10]. Activation of indoleamine 2,3-dioxygenase (IDO) diverts tryptophan metabolism toward the kynurenine (KYN) pathway, thereby decreasing serotonergic transmission and generating neuroinflammatory and neurotoxic metabolites such as quinolinic acid and 3-hydroxykynurenine [7,11].

The KYN/TRP ratio serves as an indirect biomarker of peripheral IDO activity and has been reported to be elevated in individuals with depression, schizophrenia, and those who have attempted suicide [7,12]. Accordingly, it has attracted attention as a potential indicator of both diminished serotonergic function and immune activation.

Among psychometric tools, the Beck Suicide Intent Scale (SIS, 1974) is widely used to evaluate the planning, perceived lethality, and post-attempt attitudes of individuals following a suicide attempt. The 19-item scale demonstrates high internal consistency (Cronbach’s alpha ≈ 0.85) and strong test–retest reliability [13,14]. In Turkey, its validity and reliability were established by Dilbaz et al., who reported high sensitivity and specificity in identifying individuals at risk of suicide [15].

In the present study, serum tryptophan and kynurenine levels were measured, and the KYN/TRP ratio was calculated in patients admitted to the emergency department following suicide attempts. These biochemical parameters were compared with SIS scores to investigate the potential contribution of biological markers to suicide risk assessment when used alongside psychometric tools, and to explore their possible applicability in future clinical practice.

## 2. Materials and Methods

### 2.1. Study Design and Setting

This prospective, cross-sectional, and comparative study was conducted between 13 November 2024, and 13 June 2025, in the Emergency Medicine Department of Konya City Hospital. The study aimed to evaluate serum tryptophan and kynurenine levels, as well as the KYN/TRP ratio, in individuals aged ≥ 18 years who presented to the emergency department following a suicide attempt, and to compare these biochemical parameters with SIS scores.

### 2.2. Ethical Approval and Informed Consent

The research protocol was approved by the Clinical Research Ethics Committee of Selçuk University Faculty of Medicine (Decision No: 2024/590). All procedures were performed in accordance with the principles of the Declaration of Helsinki (revised 2013). Verbal and written information about the study was provided to all participants, and written informed consent was obtained.

### 2.3. Study Population

A total of 120 participants were included, i.e., 60 cases (patients presenting after a suicide attempt) and 60 controls (healthy volunteers aged ≥ 18 years with no psychiatric or systemic illness).

Inclusion criteria:

- Age ≥ 18 years;
- Presentation to the emergency department after a suicide attempt;
- Clinically stable and conscious at the time of assessment;
- Provision of written informed consent.

Exclusion criteria:

- Altered mental status or coma;
- Severe organ failure;
- Active infection or immune deficiency;
- Pregnancy;
- Conditions preventing venous blood sampling;
- Hemolyzed or lipemic samples;
- Current use of psychiatric medications.

The control group was selected from individuals admitted to the emergency department for non-psychiatric reasons, who had fasted for ≥6 h, were confirmed to have no psychiatric disorders, and provided informed consent. Case and control groups were matched as closely as possible for age and sex distribution.

### 2.4. Blood Sampling and Storage

For all participants, 10 mL of venous blood was collected. Samples were placed into plain biochemistry tubes and centrifuged within 30 min at 3000 rpm (≈1500× *g*) for 10 min at 4 °C. Serum was aliquoted into 1.5 mL tubes and stored at −80 °C until analysis.

### 2.5. Biochemical Analysis

Serum tryptophan and kynurenine levels were measured using ELISA (Bioassay Technology Laboratory, Suzhou, China).

- Sensitivity: tryptophan 0.5 pmol/mL; kynurenine 5 µg/mL.
- Measurement range: tryptophan 5–200 pmol/mL; kynurenine 500–5000 µg/mL.
- Coefficients of variation: intra-assay < 8%; inter-assay < 10%.
- Absorbance: 450 nm, Heales MB-580 microplate reader (Shenzhen Heales Bio-Tech Co., Ltd., Shenzhen, China).

Results were expressed as pmol/mL for tryptophan and µg/mL for kynurenine. For ratio calculations, values were converted to molar units (tryptophan: pmol/mL → nmol/L; kynurenine: µg/mL → µmol/L; molecular weight = 208.21 g/mol). The KYN/TRP ratio was reported on a ×10^6^ scale.

### 2.6. Psychometric Assessment

SIS, consisting of 19 items evaluating the planning of the attempt, perceived lethality, and post-attempt attitudes, was administered to all cases. The Turkish validity and reliability study was performed by Dilbaz et al. The scale was applied within the first hour of emergency admission by two emergency medicine specialists trained in its use, with an average administration time of 15–20 min.

### 2.7. Sample Size Calculation

Sample size was calculated using G*Power 3.1 software. Based on the difference in tryptophan levels between two independent groups, assuming a medium effect size (Cohen’s d = 0.5), α = 0.05, and power = 0.85, a minimum of 59 participants per group was required. The study included 60 cases and 60 controls, achieving the target power.

### 2.8. Statistical Analysis

Categorical variables were summarized as frequency (*n*) and percentage (%), while numerical variables were expressed as median (min–max) or first and third quartiles (Q1–Q3). Normality was assessed using the Kolmogorov–Smirnov and Shapiro–Wilk tests.

Comparisons were performed using the Chi-square (χ^2^) test for categorical variables, the Mann–Whitney U test for non-normally distributed continuous variables, and Student’s *t*-test for normally distributed variables. Correlations between continuous variables were assessed using Spearman’s or Pearson’s correlation tests, as appropriate. Effect sizes were reported as Cohen’s d for Student’s *t*-test and rank biserial correlation for the Mann–Whitney U test. Multivariable analyses were performed to adjust for potential confounders including age, sex, and drug-related attempts.

All statistical analyses were performed using IBM SPSS Statistics version 22.0 (IBM Corp., Armonk, NY, USA) and Jamovi version 2.6.26, with statistical significance set at *p* < 0.05.

## 3. Results

The median age of the case group was 29 years (Min = 18, Max = 72), while that of the control group was 38 (Min = 19, Max = 81). In both groups, 66.7% were female and 33.3% were male. The demographic characteristics and baseline laboratory parameters of the case and control groups were generally comparable.

Among laboratory parameters, significant differences were observed in WBC and ALT levels. The difference in WBC was 1.15 (95% CI = 0.18–2.14, Effect size = 0.25), while the difference in ALT was 3 (95% CI = 4.13–5.00, Effect size = 0.22). The distribution of age groups and other variables is presented in Table 1.

The clinical and psychiatric characteristics of the case group are summarized in Table 2.

The most frequent method of suicide attempt was multiple drug ingestion (63.3%). A psychiatric history was present in 45.0% of cases. Following psychiatric consultation, 76.7% were advised outpatient follow-up, while 23.3% were recommended psychiatric hospitalization. The median SIS score was 8 (Q1 = 4, Q3 = 14).

In metabolite analysis, tryptophan, kynurenine, and the KYN/TRP ratio were significantly lower in the case group compared to controls (Table 3). The values of suicide subtypes and the analysis of groups showing significant differences from the control group are presented in Table 4.

Correlation analysis revealed no significant associations between SIS scores and tryptophan (*p* = 0.180), kynurenine (*p* = 0.668), or KYN/TRP ratio (*p* = 0.246). Furthermore, subgroup analyses according to psychiatric consultation recommendations (outpatient follow-up vs. hospitalization) showed no significant differences in tryptophan (*p* = 0.627), kynurenine (*p* = 0.249), or KYN/TRP (*p* = 0.848). Similarly, no significant differences were observed between patients with and without a psychiatric history for tryptophan (*p* = 0.726), kynurenine (*p* = 0.567), or KYN/TRP (*p* = 0.947).

## 4. Discussion

In this study, serum tryptophan, kynurenine, and the KYN/TRP ratio were compared between individuals who attempted suicide and healthy controls, and their associations with SIS scores were evaluated. Our findings demonstrated that tryptophan, kynurenine, and the KYN/TRP ratio were significantly lower in the case group. No significant correlations were found between SIS scores and the biochemical parameters, and subgroup analyses (e.g., presence of psychiatric history) also revealed no significant differences.

Tryptophan, the precursor amino acid for serotonin synthesis in the central nervous system, plays a critical role in mood regulation, impulse control, and behavioral modulation. Previous studies have reported markedly reduced serum tryptophan levels in patients with major depressive disorder, which may increase susceptibility to suicidal behavior [7,9]. Modai et al. found significantly lower tryptophan and serotonin levels in patients with major depression who had attempted suicide [16]. Similarly, Bradley et al. reported approximately 40% lower tryptophan levels and 40% higher KYN/TRP ratios in adolescents with depression, suggesting an association with suicidal ideation [17]. In line with these studies, our results showing significantly reduced tryptophan levels in cases support the hypothesis that serotonergic deficiency may contribute to the neurobiological basis of suicidal behavior.

Kynurenine, a metabolite of tryptophan via indoleamine 2,3-dioxygenase (IDO), is elevated under conditions of inflammation, stress, or immune activation. Sublette et al. reported significantly higher plasma kynurenine levels in depressive patients with a history of suicide attempts and identified a positive correlation between the KYN/TRP ratio and neopterin levels [7]. Myint et al. proposed that increased IDO activity diverts tryptophan metabolism toward the kynurenine pathway, leading to the accumulation of potentially neurotoxic metabolites [18]. In contrast, in our study both kynurenine and the KYN/TRP ratio were lower, which may reflect the absence of measurable inflammatory activity at the time of assessment, the exclusion of patients on psychotropic medications, methodological limitations of ELISA compared with gold-standard techniques, or insufficient sample size to detect small-to-moderate effects.

The lack of significant correlations between SIS scores and tryptophan, kynurenine, or the KYN/TRP ratio suggests that biomarkers alone may not adequately reflect suicide risk. While some studies have reported associations between the KYN/TRP ratio and suicidal ideation, others have failed to demonstrate such links between biochemical markers and psychometric measures [17]. For instance, Nettis et al. observed elevated KYN/TRP ratios in patients with treatment-resistant depression and suicidal ideation [19]. These findings highlight the complex interplay of biological, psychosocial, environmental, and cognitive factors in suicidal behavior. Recent evidence also suggests that tryptophan metabolism may shift toward the kynurenine pathway through microglial activation and neuroinflammation in individuals with depression and suicidality [20,21]. Thus, evaluating both peripheral serum levels and central neuroinflammatory activity is essential for a more comprehensive understanding.

The KYN/TRP ratio primarily reflects IDO activity, which catalyzes the rate-limiting step in tryptophan degradation and is upregulated in response to inflammatory and stress-related stimuli. Previous studies have reported elevated kynurenine levels and KYN/TRP ratios in patients with major depression and suicide attempts, suggesting enhanced IDO activity [22]. Both animal and clinical studies demonstrate that chronic stress can upregulate IDO, shifting tryptophan metabolism toward kynurenine and leading to the accumulation of neurotoxic metabolites such as quinolinic acid (QUIN), which are thought to contribute to depressive symptoms and suicidal behavior [23].

In our study, the reductions observed in biochemical parameters may indicate altered metabolic balance in patients after a suicide attempt. However, these biomarkers alone do not possess diagnostic or prognostic value; rather, they may contribute to risk stratification when used in conjunction with psychometric scales such as SIS and inflammatory markers (e.g., IL-6, CRP).

## 5. Limitations

This study has several limitations. First, it was conducted in a single center with a relatively small sample size, and its cross-sectional design does not allow causal inference. In addition, inflammatory markers (IL-6, TNF-α) were not measured, and other downstream metabolites of tryptophan, such as quinolinic acid and kynurenic acid, were not assessed. The ELISA method used for biochemical analysis has lower sensitivity compared with the gold-standard LC-MS/MS technique. Furthermore, the predominance of drug ingestion, particularly multiple drug intake, as the method of suicide among participants may represent a potential confounder influencing biochemical parameters.

## 6. Conclusions

This study demonstrated that serum tryptophan, kynurenine, and the KYN/TRP ratio were significantly lower in patients presenting to the emergency department after a suicide attempt compared with healthy controls. No significant associations were found between these biochemical markers and SIS scores, nor were there differences in subgroup analyses based on psychiatric history or psychiatric consultation recommendations. Interestingly, while previous literature frequently reports increased tryptophan degradation and activation of the kynurenine pathway in suicidal individuals, our study identified lower kynurenine levels. This finding suggests that, in the context of suicidal behavior, tryptophan metabolism may shift not only toward the kynurenine pathway but also toward alternative metabolic routes. Therefore, our study provides a novel perspective compared with prior KYN/TRP research and contributes valuable insight to the literature. Overall, reduced levels of tryptophan, kynurenine, and KYN/TRP may serve as complementary biomarkers alongside psychometric assessments; however, their precise role in clinical practice remains uncertain. Larger, multicenter studies with extended follow-up are warranted to validate these findings and clarify their potential clinical utility.

## Figures and Tables

**Table 1:** Demographic and General Characteristics of Suicide Attempt Cases (*n* = 60) and Control Group (*n* = 60).

Variables			Case	Control	*p*
Gender	Male	*n* (%)	20 (33%)	20 (33%)	
	Female	*n* (%)	40 (66.7%)	40 (66.7%)	
Age groups	18–29	*n* (%)	28 (46.7%)	20 (33.7%)	*p* = 0.38 x^2^ = 3.16
	30–45	*n* (%)	21 (35.0%)	22 (36.7%)	
	46–59	*n* (%)	6 (10.0%)	11 (18.3%)	
	≥60	*n* (%)	5 (8.3%)	7 (11.7%)	
Hematologic Parameters	WBC (10^3^ µL)	Median (Min–Max)	8.7 (4.4–21.1)	7.0 (3.2–20.0)	*p* = 0.02 U = 1357.0
	Hgb (g/dL)	Mean ± SD	12.8 ± 2.1	13.2 ± 2.1	*p* =0.35 t = 0.10
	PLT (10^3^ µL)	Mean ± SD	243 ± 59	270 ± 66	*p* = 0.08 t = 2.35
Biochemical Parameters	Glucose (mg/dL)	Median (Min–Max)	91 (70–244)	96 (67–224)	*p* = 0.19 U = −1547.5
	Creatinine (mg/dL)	Median (Min–Max)	0.67 (1.12)	0.71 (0.47–1.38)	*p* = 0.24 U = 1577.5
	AST (U/L)	Median (Min–Max)	19 (10–68)	17 (5–51)	*p* = 0.07 U = 1454.5
	ALT (U/L)	Median (Min–Max)	15 (2–84)	18 (8–41)	*p* = 0.04 U = 1404.5
	CRP (mg/dL)	Median (Min–Max)	1.8 (0.6–40.4)	1.6 (0.5–66.0)	*p* = 0.69 U = 1724.5
Coagulation Panel	INR	Mean ± SD	1.09 ± 0.8	1.05 ± 0.09	*p* = 0.38 t = −2.13
	aPTT (s)	Median (Min–Max)	23.4 (18.7–38.3)	22.8 (18.7–27.8)	*p* = 0.26 U = 661

Min: Minimum, Max: Maximum, WBC: White Blood Cell, Hgb: Hemoglobin, PLT: Platelet, AST: Aspartate transaminase, ALT: Alanine aminotransferase, CRP: C-reactive protein, U: Mann–Whitney U, t: Student-t Test, X^2^: Chi-Square.

**Table 2:** Other Characteristics of Suicide Attempt Cases (*n* = 60).

Variable		*n*	%
Suicide method	Single drug intake	15	25.0
	Multiple drug intake	38	63.3
	Other	7	12.7
Psychiatric History	Yes	27	45.0
	No	33	55.0
Psychiatric Consultation Recommendations	Outpatient Follow-up	46	76.7
	Psychiatric Admission	14	23.3
		**Median**	**Q1–Q3**
SIS		8	4–14
Systolic Blood Pressure (mmHg)		107	100–120
Diastolic Blood Pressure (mmHg)		70	65–80
Pulse (bpm)		84	76–86
SpO_2_ (%)		96	95–97

Q: Quartile, SIS: Beck Suicide Intent Scale.

**Table 3:** Cases (*n* = 60).

Variable		Case	Control	p	M.Dif.	95% CI		Effect Size
						Lower	Upper	
Tryptophan (µmol/mL/10^6^)	Median (Min–Max)	35.9 (21.4–154.4)	54.4 (23.9–147.6)	0.002 (U = 1220)	2.1	2.4	2.8	0.32
Kynurenine (µg/mL)	Median (Min–Max)	1534.5 (1014.7–2837.1)	2384.0 (1612.4–5253.1)	<0.001 (U = 281)	845	659	1056	0.84
KYN/TRP (×10^6^)	Mean ± SD	40.9 ± 16.2	48.8 ± 20.8	0.02 (t = 2.31)	7.8	1.1	14.6	0.42

M.Dif: Mean Differences, CI: Confidence Interval.

**Table 4:** Tryptophan, Kynurenine, and KYN/TRP values and statistical comparisons according to subgroups of suicide cases.

		Subgroups of Suicide Cases			
Variable		Single Drug Intake	Multiple Drug Intake	Other	*p* *
Tryptophan (µmol/mL/10^6^)	Median (Min–Max)	34.9 (24.5–121.6)	36.3 (21.4–154.4)	35.3 (27.2–79.4)	*p* ^bc^ = 0.04
Kynurenine (µg/mL)	Median (Min–Max)	1560.7 (1014.7–2314.8)	1544.9 (1226.5–2837.1)	1270.2 (1052.4–1983.4)	*p* ^ac^ < 0.01 *p* ^bc^ < 0.01 *p* ^xc^ < 0.01
KYN/TRP (×10^6^)	Mean ± SD	40.2 ± 15.8	44.4 ± 15.9	29.8 ± 19.5	*p* ^ac^ = 0.04

* a: Single drug intake, b: Multiple drug intake, x: Other, c: Control.

## Data Availability

The data that support the findings of this study are available from the corresponding author upon reasonable request.
